# Integrating Experimental Physicochemical Data with PBPK Modeling to Guide the Translational Development of the Antifungal 1,3,4-Oxadiazole Derivative LMM6

**DOI:** 10.1021/acsomega.6c00258

**Published:** 2026-07-02

**Authors:** João Marcos Barbosa Piai, Victor Nery Machado Lippa, Edilainy Rizzieri Caleffi-Marchesini, Fernanda Belincanta Borghi Pangoni, Maria Sueli Felipe, Erika Seki Kioshima, Andrea Diniz

**Affiliations:** † State University of Maringá (UEM), Maringá, Paraná 87020-900, Brazil; ‡ Ingá University Center (UNINGÁ), Maringá, Paraná 87020-900, Brazil; § 28106 Catholic University of Brasilia and University of Brasília, Federal District, Brasília 70910-900, Brazil

## Abstract

The rising mortality of invasive fungal infections highlights an urgent need for novel antifungal agents with improved translational profiles. The 1,3,4-oxadiazole derivative LMM6 has emerged as a promising candidate. Therefore, this study aimed to build a comprehensive biopharmaceutical profile of LMM6 and apply PBPK modeling as a rational tool in early drug development to evaluate its pharmacokinetic feasibility in mice and humans. In vitro biopharmaceutical experiments assessed solubility (shake-flask method) in compendial and biorelevant media, alongside experimental logP determination. ADME data were generated in silico and integrated into PBPK models. LMM6 exhibited a logP of 2.11 and low thermodynamic solubility across all media, though values slightly improved under acidic conditions and in the presence of bile salts. Mouse simulations (5 mg/kg q12h) predicted a plasma Cmax of ≈7.3 mg/L and an intracellular Cmax of 2.77 mg/L (kidney) and 1.21 mg/L (spleen), all below the minimum inhibitory concentration (MIC; 8–32 mg/L), with rapid declines and negligible troughs (Cmin < 0.1 mg/L). These findings suggest that previously reported mouse efficacy may not be fully explained by systemic exposure. Conversely, human PBPK extrapolation (700 mg q12h) predicted a more favorable profile, with interstitial renal and splenic concentrations reaching the MIC range from the third dose onward. While these in silico simulations suggest human exposures could surpass those in mice, these results remain hypothesis-generating and require further preclinical and clinical validation. This integrated approach provides critical translational insights for prioritizing formulation optimization and dosing strategies for this promising antifungal candidate.

## Graphical abstract

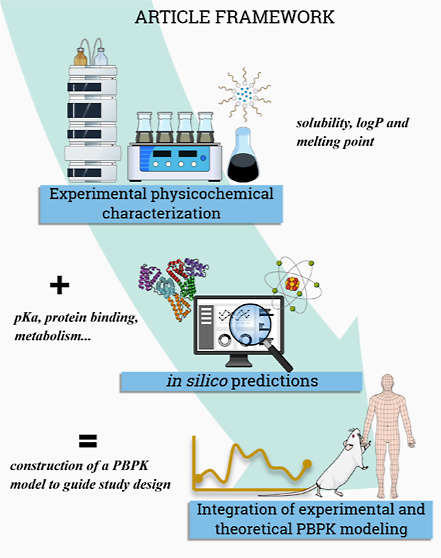

## 1 Introduction

The increasing incidence and high mortality of invasive fungal infections highlight an urgent need for novel antifungal agents. Current therapeutics are constrained by limited spectra of activity, dose-limiting toxicity, and the emergence of resistance, which together restrict clinical options and motivate the search for leads with improved translational profiles.^1–3^ The 1,3,4-oxadiazole derivative LMM6 (Figure 1) has shown promising antifungal activity in preliminary in vitro and in vivo studies against Candida spp. and Paracoccidioides spp., with minimum inhibitory concentration (MIC) values against Candida spp. of 8–32 mg/L,^4,5^ positioning it as a candidate for further preclinical development. However, progression toward clinical evaluation requires an integrated understanding of the compound’s physicochemical properties and how these influence formulation, absorption, and tissue distribution.^6,7^

**Figure 1:**
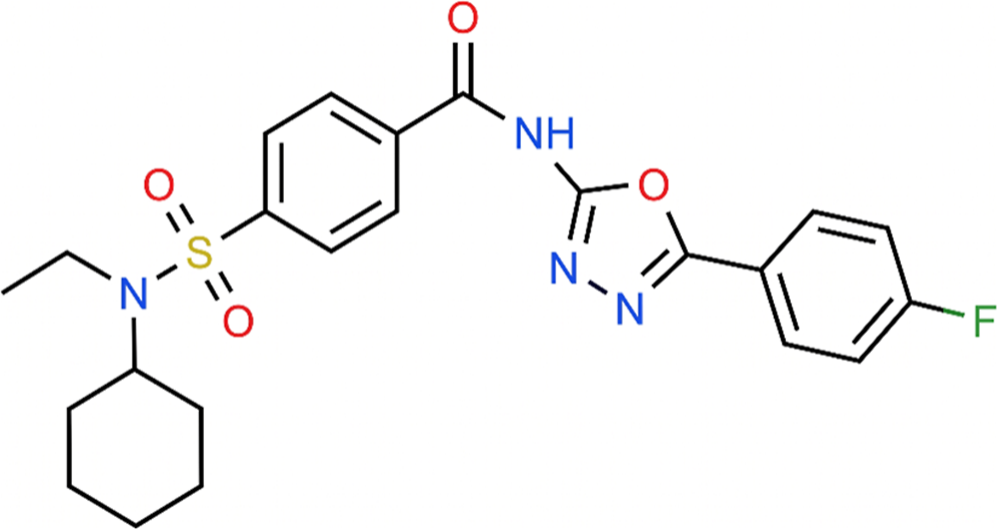
Chemical structure of the substance 4-[cyclohexyl­(ethyl)­sulfamoyl]-N-[5-(4 fluorophenyl)-1,3,4-oxadiazol-2-yl]­benzamide (LMM6).

Key physicochemical attributes—such as aqueous solubility, lipophilicity (logP), and thermal behavior—directly impact a drug’s dissolution, oral bioavailability, and ability to reach therapeutic concentrations at target sites, including the intracellular compartments where many fungal pathogens reside.^8–10^ For poorly soluble or highly lipophilic compounds, formulation and dosing strategies must be carefully designed. Suboptimal design can lead to subtherapeutic exposures, risking treatment failure and drug resistance. Conversely, it may cause off-target toxicity, limiting the drug’s safety margin.^11,12^ Therefore, the early experimental characterization of these parameters, combined with predictive in silico tools, is crucial for strengthening decision-making during lead optimization.^13,14^

Model-informed drug discovery and development (MID3) approaches, such as physiologically based pharmacokinetic (PBPK) modeling, facilitate the integration of experimental physicochemical data with biological parameters to predict tissue exposures.^15,16^ Recently, a hybrid PBPK model was proposed to accelerate this process. This novel approach integrates mechanistic relationships, in vitro parameters, and PBPK modeling into the final stages of the drug discovery pipeline, generating richer data for lead candidates.^17^

However, building a PBPK model traditionally requires a large number of inputs. In the context of novel drug discovery, where experimental data are often limited, in silico predictions serve as valuable parameters.^14,18^ For LMM6, this study aimed to develop a comprehensive biopharmaceutical profile by integrating experimental in vitro data and previously reported in vivo efficacy against fungal infections in mouse target tissues (kidney and spleen)^4^ with in silico ADME predictions. Using Hybrid PBPK modeling, we explored the pharmacokinetic profile of LMM6 in mice and humans to evaluate its translational potential, highlighting the use of PBPK modeling as a strategic tool to guide the rational development of novel drugs.

## 2 Materials and Methods

### 2.1 Materials

The pure drug substance of LMM6 was obtained from Life Chemicals (Batch F2832-016) (Life Chemicals Inc. Niagara-on-the-Lake, Canada). All chemicals used for media preparation and sample analysis were of analytical or high-performance liquid chromatography (HPLC) grade and were commercially purchased from various suppliers: sodium hydroxide (Anidrol, Diadema, Brazil), dichloromethane (Honeywell International Inc., Muskegon, USA), trifluoroacetic acid, acetonitrile, glyceryl monooleate, sodium oleate (Merck KGaA, Darmstadt, Germany), sodium taurocholate, pepsin (Inlab, São Paulo, Brazil), sodium chloride (Biotec, Pinhais, Brazil), 1-octanol, maleic acid (Sigma-Aldrich, Darmstadt, Germany), hydrochloric acid, glacial acetic acid, potassium phosphate monobasic (LabSynth, São Paulo, Brazil), and lecithin (Alfa Aesar, Massachusetts, USA). Ultrapure water was produced using the ORU-2E purification system (Union, São José dos Pinhais, Brazil).

### 2.2 Softwares Used

Physicochemical and pharmacokinetic properties of LMM6 were predicted in silico using ADMET Predictor software (v. 11, SimulationPlus, Lancaster, CA, USA). The estimates generated by this tool served as input parameters for the construction and calibration of PBPK models. The physiologically based pharmacokinetic (PBPK) models for both mouse and human simulations were developed using PK-Sim software (version 12), which is part of the Open Systems Pharmacology Suite platform (Bayer Technology Services, Leverkusen, Germany). Additionally, all statistical analyses and the one-way analysis of variance (ANOVA) were performed using Microsoft Excel software (Microsoft Corp., Redmond, WA, USA) with the Analysis ToolPak add-in.

### 2.3 Media Composition

Plain buffers, including hydrochloric acid 0.1 M (pH 1.2), acetate buffer (pH 3.0 and 4.5), and monobasic potassium phosphate buffer 0.05 M (pH 7.4), were prepared following the guidelines outlined in the USP.^19^ For biorelevant media, Fasted State Simulated Gastric Fluid (FaSSGF-v2) and Fasted State Simulated Intestinal Fluid (FaSSIF-v2) were prepared based on established literature protocols.^20–25^ The FaSSIF-V2 blank (maleate buffer) was also prepared using the components of FaSSIF-v2, excluding lecithin and sodium taurocholate.^21^

Additionally, fed state simulated intestinal fluid (FeSSIF-v2) was formulated in accordance with previously published protocols.^26,27^ During the dichloromethane evaporation steps in the preparation of FeSSIF-v2, a modification to the procedure was made. Instead of using a rotary evaporator,^26^ a Christ RVC 2-25 CDplus speed vacuum concentrator was utilized. The updated protocol retained a 30 min evaporation duration for each step, at a speed of 1300 rpm and room temperature, until visual confirmation of dichloromethane evaporation was achieved.^28,29^

### 2.4 Liposolubility Characterization

One way to assess this property is by comparing the distribution behavior of the molecule between the aqueous and the organic phases. To characterize the liposolubility of LMM6, a shake-flask method was employed. Acetate buffer at pH 4.5 was used to ensure that only the nonionizable form of the molecule was present in the medium, based on theoretical predictions that estimate a p*K*_a_ of 6.66 (ADMET Predictor v.11). This method has been adapted for the use of microvolumes.^30,31^

Initially, both blank solvents (acetate buffer at pH 4.5 and *n*-octanol) were mutually saturated at 37 °C for 24 h in a mechanical shaker (Thoth 6420B, Thoth Equipamentos, Piracicaba, Brazil), followed by a resting period to allow phase separation. Three stock solutions of LMM6 in *n*-octanol were prepared, each with a concentration of 0.200 mg/L.^30,31^

Subsequently, using the LMM6 stock solution, three sets of analyses were prepared by varying the proportions of the organic and aqueous phases (organic, intermediate, and aqueous). In the first set (intermediate), equal volumes of both solvents were mixed, with 2 mL of LMM6 stock solution in *n*-octanol and 2 mL of acetate buffer (pH 4.5). In the second set (organic), the organic phase was doubled, consisting of 4 mL of LMM6 stock solution in *n*-octanol and 2 mL of acetate buffer. Finally, in the third set (aqueous), the aqueous phase was doubled, with 2 mL of LMM6 stock solution and 4 mL of acetate buffer. All solutions were prepared in duplicate. The samples were then shaken in an orbital mechanical shaker for 1 h at 37 °C, followed by centrifugation for 10 min at 2300 rpm. After centrifugation, aliquots of the stock solutions, as well as the aqueous and organic phases of the samples, were analyzed by HPLC using a previously validated method (Section 2.7).^30,32^

### 2.5 Melting Point Determination

The melting point of LMM6 was determined using the Labstore PF 1500 equipment (Labstore, Brazil). Initially, the drug was pulverized and sieved to ensure homogeneity. Each sample was inserted into glass capillaries and placed in the heating block of the equipment. Heating was carried out at a constant rate of approximately 1–2 °C per minute, while the samples were visually monitored for the detection of melting. The melting temperatures were recorded, and the procedure was repeated three times to ensure the accuracy of the results. Finally, the average melting point values, along with the corresponding standard deviations, were calculated.^33^

### 2.6 Instruments and Chromatographic Conditions

The HPLC analytical method was developed and adapted from two studies, which utilized a 1,3,4-oxadiazole derivative and itraconazole,^34,35^ molecules structurally like LMM6, as no validated method for this molecule is available in the literature. The HPLC analyses were carried out using a Shimadzu LC-20AT system (Shimadzu Corporation, Tokyo, Japan) with a UV–vis SPD-20A detector set at a wavelength of 273 nm, a DGU-20A5 degasser, a CBM-20A controller, and a Phenomenex Inertsil 5 μm ODS-2, C18 column (150 × 4.6 mm) (GL Sciences, California, USA).

The analyses were conducted using an isocratic elution. The mobile phase consisted of acetonitrile and ultrapurified water (80:20 v/v) with 0.1% (v/v) trifluoroacetic acid. The run time was 5 min, with a flow rate of 0.800 mL/min at a column temperature of 30 °C. The injection volume was 20 μL, and data integration was performed using LC Solution software (Shimadzu Corporation, Tokyo, Japan).

### 2.7 Analytical Method Validation

A chromatographic method for HPLC was validated for the determination of LMM6, following the guidelines established by the International Conference on Harmonization (ICH Q2-R1)^36^ and RDC N° 166 ^37^. Parameters such as system suitability, specificity, linearity, sensitivity, precision, accuracy, limit of detection (LOD), and limit of quantification (LOQ) were assessed. Statistical significance was determined with a p-value of less than 0.05 using ANOVA. Data manipulation and statistical analysis were performed using Excel Software (Microsoft, Washington, USA).

To evaluate method specificity, we analyzed and compared the retention times of LMM6 with those of interfering peaks. Since no official compendial standard is available for this novel molecule, the pure LMM6 substance (Life Chemicals, batch F2832-016) was used as the working standard. The stock standard solution was prepared by dissolving the pure LMM6 in acetonitrile to achieve a concentration of 1000.0 mg/L.

Linearity was assessed by constructing three analytical curves with standard solutions of LMM6 at seven different concentration ranges from 2.0 to 20.0 mg/L. Linear regression was applied to analyze the peak area data versus the LMM6 concentration. The data obtained underwent regression analysis using the least-squares method with a weighting factor of 1/x. Normality and homoscedasticity were evaluated using the Grubbs test^38^ and visual inspection of standardized residuals. The limit LOD and LOQ were determined using eqs 1 and ([Disp-formula eq2]), respectively, based on the standard error and angular coefficient obtained during validation^36^

1 $$LOD=3.3^{*}\left(\frac{s\tan dard\;error}{angular\;coefficient}\right)$$

2 $$LOQ=10^{*}\left(\frac{s\tan dard\;error}{angular\;coefficient}\right)$$

Method precision was estimated at two levels: repeatability (intraday precision) and intermediate precision (interday precision). Nine determinations, comprising three concentrations with three replicates each, were performed within the linear range. Precision was expressed as the coefficient of variation (CV %). The acceptance criterion was a CV % of less than 5.0%, considering the method’s intrinsic variability and working sample concentration. For accuracy analysis, triplicate LMM6 standard solutions were prepared at three different concentration levels (6.0, 12.0, and 18.0 mg/L). The acceptance criterion was a recovery range of 95.0% to 105.0%, in accordance with the precision requirements.^36,37^ Statistical evaluation was conducted using the ANOVA test to compare the means of independent samples.

### 2.8 Solubility Assessment

To ensure the achievement of equilibrium solubility, an excess of solid LMM6 (approximately 1 mg per analysis) was weighed for each experiment. This amount was chosen to exceed the solubility values previously predicted in silico for water, FaSSGF-v2, FaSSIF-v2, and FeSSIF-v2 by ADMET Predictor v.11 (SimulationPlus, Lancaster, CA, USA), as no prior experimental data were available for this molecule. The detailed results of the in silico solubility simulations are provided in the Supporting Information (Table S1).

The software did not provide predictions for phosphate and HCl media; therefore, we relied on predictions for other media. Samples were prepared in triplicate, and the media evaluated separately included 1.0 mL of HCl (pH 1.2), phosphate buffer (pH 7.4), FaSSGF-v2 (pH 1.6), FaSSIF-v2 (pH 6.5), FeSSIF-v2 (pH 5.8), and ultrapure water at pH 6.0. These components were added to create saturated solutions in 1.5 mL of microcentrifuge tubes.

The solubility equilibrium state is established when a solution becomes saturated in the presence of excess solid, ensuring a dynamic balance between the dissolved solute and the undissolved solid phase.^39^ The solubility tests of LMM6 were conducted using the shake-flask technique with an orbital shaker (Thoth 6420B, Thoth Equipamentos, Piracicaba, Brazil). The samples were gently shaken to remove bubbles and stored on the orbital shaker maintained at 37 °C, rotating at 200 rpm for 24 h. Afterward, all samples were centrifuged using a refrigerated centrifuge (Hermle Z36HK, Hermle Labortechnik GmbH, Wehingen, Germany) at 25 °C, at 12,000 rpm for 10 min to sediment the excess nonsolubilized LMM6. This method was adapted from pharmacopoeial techniques, utilizing microvolumes.^39–41^ The samples were analyzed by HPLC using the previously developed and validated method (Section 2.7).

Because of the low solubility of LMM6 and the method requirements for HPLC analysis, samples in HCl, phosphate buffer, acetate buffer, maleate buffer, and water were concentrated. This was done by removing a small portion of the supernatant and using a speed vacuum concentrator (Christ RVC 2–25 CDplus, Martin Christ Gefriertrocknungsanlagen GmbH, Osterode am Harz, Germany).^28,29^

### 2.9 Physiologically Based Pharmacokinetic Modeling and Simulation

The PBPK models were developed and parametrized within the PK-Sim environment (version 12), as previously mentioned in Section 2.1, following standard best practices and protocols.

The model was constructed using a bottom-up approach, integrating physicochemical parameters experimentally obtained in this study with in silico-predicted data, as presented above. Species-specific physiological databases for both mice and humans were incorporated into the modeling environment; these resources are publicly available from the Open-Systems-Pharmacology GitHub releases (v3.0.1).

For model parametrization, experimental data on solubility in compendial media and partition coefficient were used. Parameters not experimentally available, such as p*K*_a_ and plasma protein binding, were estimated through theoretical prediction using ADMET Predictor software (v.11, SimulationPlus, California, USA). All input parameters used for model construction in PK-Sim are listed in Table 1.

**Table 1:** Physicochemical and Biological Parameters of LMM6 Used in the Model

Parameters	Value	Reference
physicochemical		
molecular weight (g/moL)	272.54	ADMET predictor
LogP	2.11	experimental
p*K*_a_ (acid)	6.66	ADMET predictor
fraction unbound mouse	0.047	ADMET predictor
fraction unbound human	0.036	ADMET predictor
partition coefficient	PK-Sim standard	PK-Sim
cellular permeability	PK-Sim standard	PK-Sim
specific intestinal permeability	2.24 × 10^–06^ cm/min	PK-Sim
solubility (ph 1.2–7.4)	2.95–4.94 mg/L	experimental
metabolism		
in vitro metabolic rate in the presence of recombinant CYPs/Michaelis–Menten		
CYP3A4 Km	174.52 μM	ADMET predictor
CYP2C19 Km	119.31 μM	ADMET predictor
CYP2C9 Km	6.37 μM	ADMET predictor

Initially, a PBPK model was developed for the mice. The simulated administration protocol was based on the dosing regimen used in the study by Faria et al., consisting of a 5 mg/kg dose administered by an intravenous (IV) bolus every 12 h for 5 days. Additionally, single IV dose simulations and dose extrapolation studies were conducted to assess the pharmacokinetic linearity (5 to 100 mg/kg). The simulated concentrations in the tissues of interest (kidneys and spleen) were compared with the in vitro observed MIC values (8 to 32 mg/L) to assess the potential for achieving therapeutic levels.^4^

The individual physiological parameter was set to the default body weight values provided by PK-Sim for mouse (0.020 kg). Following the verification of the animal model, an extrapolation to a human PBPK model was performed using the platform’s interspecies scaling functionalities to predict the pharmacokinetic profile in humans as single and multiple doses. The physiological parameters assumed for the European individual were gender male, age 30 years old, weight 73 kg, height 176 cm, and body mass index 23.53 kg/m^2^.

Simulations of plasma and tissue exposure of LMM6 for humans after IV administration of a dose of 350 mg twice a day (BID) were initially explored to get the minimal dose able to achieve the MIC concentrations in plasma and tissues.

In parallel, oral administration models for both mice and humans were developed using the same software to characterize the compound’s oral biopharmaceutical behavior. For these simulations, the formulation was defined as “dissolved” within the PK-Sim software, and the simulated oral dosing regimen mirrored the protocol by Faria et al. (5 mg/kg dose administered every 12 h for 5 days).

## 3 Results

### 3.1 Analytical Method Validation

The specificity of the developed HPLC method was confirmed by analysis of the LMM6 standard solution. A representative chromatogram, obtained with a UV–vis detector at 273 nm, is now provided in the Supporting Information (Figure S1). Chromatograms of the mobile phase, diluent, LMM6 standard, pharmacopeial buffers, and biorelevant media showed no interfering peaks at the retention time of LMM6, confirming the specificity.

Sensitivity was evaluated through LOD and LOQ, determined using equations based on the standard error and slope from the validation process (eq 1 and 2). LMM6 showed an LOD of 0.28 mg/L and an LOQ of 0.85 mg/L. Linearity was assessed from the LOQ, with a strong correlation between peak areas and LMM6 concentrations across the tested range. The regression eq (eq 3) yielded a determination coefficient (*R* ^2^ = 0.998) and a correlation coefficient (*R* = 0.999), confirming linearity.

3 y=88080×x−4807.1

ANOVA confirmed model adequacy (Table 2). The lack-of-fit test showed an F-value of 2.18, below the tabulated value of 3.07, indicating no significant deviation from linearity. The regression was statistically significant, with an *F*-value of 24,617.95 exceeding the critical value of 4.38. Grubbs’ test^38^ revealed no outliers, and residual analysis supported homoscedasticity (Figure 2).

**Table 2:** ANOVA Results for Linearity: Significance of Regression and Lack of Fit Analysis

parameter	degrees of freedom	sum of squares	mean square	*F* value	F _tab_
regression	1	6.25 × 10^12^	6.25 × 10^12^	24617.95	4.38
residual	19	4.83 × 10^9^	2.54 × 10^8^		
lack off it	5	2.96 × 10^9^	5.92 × 10^8^	2.18	3.07
pure error	14	3.80 × 10^9^	2.71 × 10^8^		

**Figure 2:**
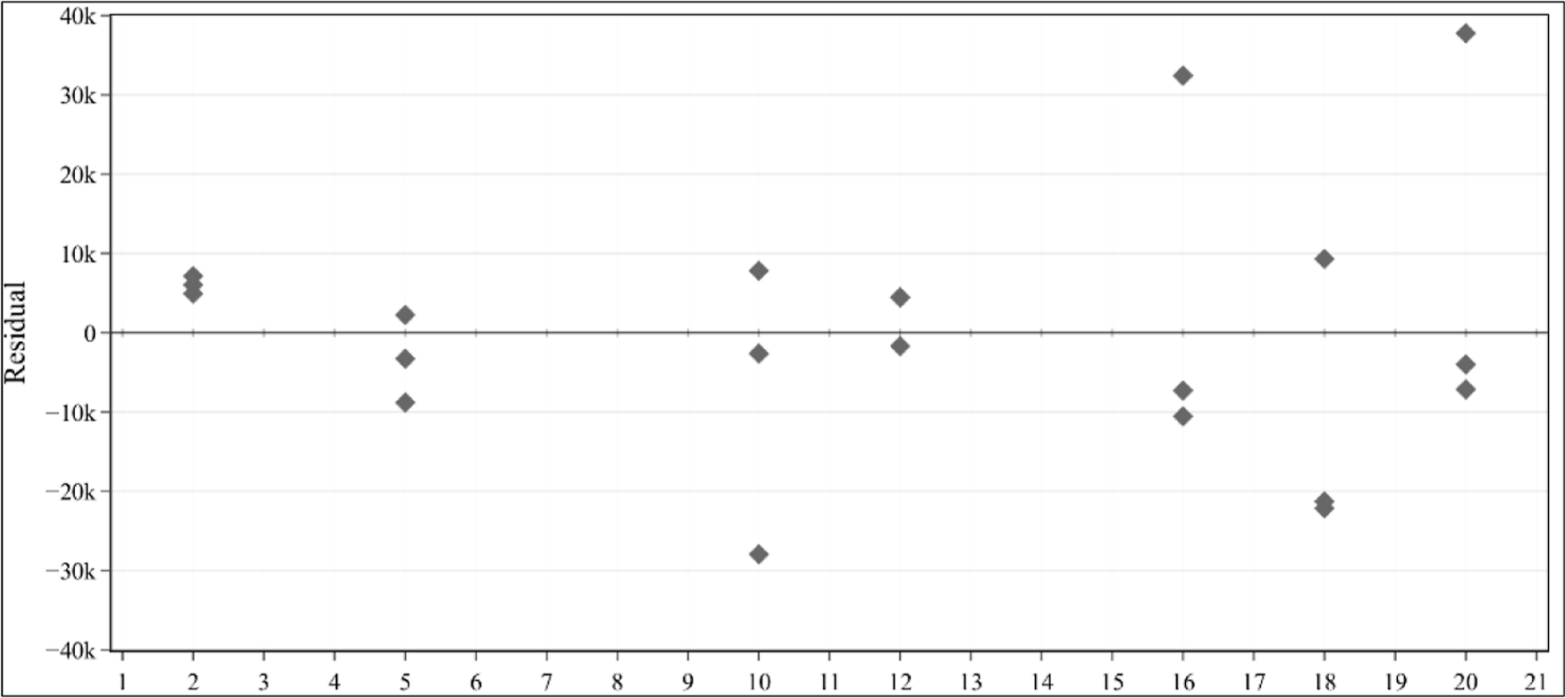
Studentized residuals dispersion analysis from the LMM6 linearity test.

Precision was demonstrated by intra- and interday analyses at 6.0, 12.0, and 18.0 mg/L, with RSD values below 5.0% (Table 3). Accuracy at the same levels showed mean recoveries of 96.40% (±0.87%), 98.88% (±2.53%), and 97.07% (±0.67%), all within the 95.0–105.0% acceptance range (Table 4).^36,37^

**Table 3:** Intraday and Interday Precision Results for the LMM6 Molecule

	analyst	theoretical concetration (mg/L)	experimental concetration (mg/L)	RSD (%)
intraday	1	6	5.78 ± 0.05	0.87
		12	11.86 ± 0.30	2.53
		18	17.47 ± 0.11	0.67
interday	2	6	6.01 ± 0.07	1.20
		12	12.36 ± 0.11	0.95
		18	18.18 ± 0.14	0.81
	-	6	5.89 ± 0.13	2.32
comparative		12	12.11 ± 0.33	2.80
		18	17.82 ± 0.41	2.29

**Table 4:** Recovery Results for the LMM6 Molecule

theoretical concetration(mg/L)	experimental concetration (mg/L)	recovery (%)
6	5.78 ± 0.05	96.40 ± 0.87
12	11.86 ± 0.30	98.88 ± 2.53
18	17.47 ± 0.11	97.07 ± 0.67

### 3.2 Melting Point Determination

Melting point measurements were conducted in triplicate. The mean melting temperature for LMM6 was 222.53 °C (±0.87 °C), obtained from three independent determinations.

### 3.3 Liposolubility Characterization

The experimentally determined logarithm of the partition coefficient (log P) was 2.11 (Table 5). The logP values from the six replicates showed a difference of less than 0.3 logarithmic units.^30,31^

**Table 5:** Results of logP Analysis of LMM6

sample		organic/aqueous ratio (log^10^)	average	average log P
aqueous	1	2.11	2.14	
	2	2.18		2.11
intermediate	1	2.16	2.19	
	2	2.23		
organic	1	1.99	1.99	
	2	1.99		

### 3.4 Experimental Solubilities Assessment

The thermodynamic solubility of LMM6 after 24 h at 37 °C was determined in pharmacopeial and biorelevant media, including HCl 0.1 M (pH 1.2), acetate buffer (pH 3.0 and 4.5), ultrapure water (pH ∼ 6.0), phosphate buffer (pH 7.4), FaSSGF-V2 (pH 1.6), FaSSIF-V2 (pH 6.5), and FeSSIF-V2 (pH 5.8). Measured solubility values (mean ± SD) are presented in Table 6.

**Table 6:** Experimental Thermodynamic Solubility of LMM6

media	solubility (mg/L)
HCl 0.1 M pH 1.2	4.94 ± 0.06
acetate buffer pH 3.0	4.34 ± 0.04
acetate buffer pH 4.5	4.31 ± 0.06
water pH 6.0	2.93 ± 0.04
phosphate buffer pH 7.4	2.95 ± 0.06
FaSSGF-V2 pH 1.6	12.95 ± 0.34
FaSSIF-V2 pH 6.5	3.80 ± 0.07
FaSSIF-V2 pH 6.5 (blank)	2.70 ± 0.06
FeSSIF-V2 pH 5.8	5.13 ± 0.16

Overall, LMM6 showed consistently low solubility across all of the tested media. Solubility was approximately 3-fold higher under gastric (acidic) than intestinal (neutral/basic) conditions in both compendial and biorelevant media. While differences in sodium taurocholate concentrations and pH generated distinct solubility values among FaSSGF-V2, FaSSIF-V2, and FeSSIF-V2, the absolute maximum solubility remained low (<13 mg/L). Although solubility in FeSSIF-V2 (fed state) was slightly higher than that in FaSSIF-V2 (fasted state), both values reflect a poor solubility profile. The relationship between log S and pH is illustrated in Figure 3.

**Figure 3:**
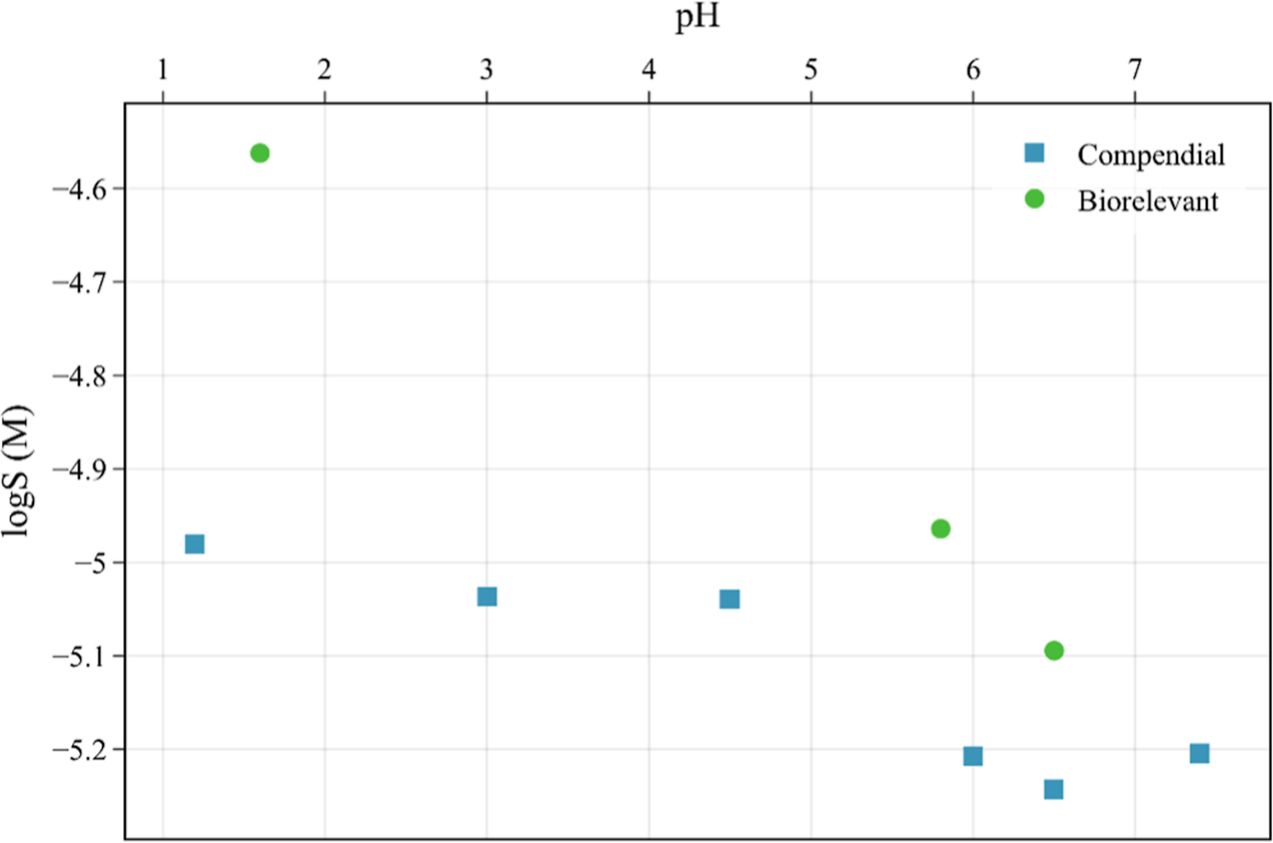
Solubility-pH profile for LMM6. Blue squares are correspondent to compendial media and green circle are correspondent to the biorelevant media.

A two-way ANOVA (details in the Supporting Information) revealed significant correlations of solubility with both bile salt concentration (p = 1.09 × 10^–4^) and pH (p = 3.03 × 10^–10^).

### 3.5 Physiologically Based Pharmacokinetic Modeling and Simulation

A bottom-up PBPK model was developed in PK-Sim by combining experimentally measured inputs (solubility, log P) with in silico predictions for parameters p*K*_a_, plasma–protein binding, and metabolic clearance, as presented in Table 1. Simulations for the oral route were performed to characterize the compound’s biopharmaceutical behavior, predicting a fraction absorbed (*F*_abs_) of ≈0.006 (0.6%) in mice and ≈0.003 (0.3%) in humans, which resulted in negligible systemic and tissue concentrations.

In the mouse intravenous simulations (Figure 4A), the predicted peak plasma concentration (Cmax) after the second dose was approximately 7.29 mg/L, which remains below the in vitro minimum inhibitory concentration (MIC; 8–32 mg/L). Analysis of target tissues revealed that all compartments likewise remained below the in vitro MIC reported: predicted total intracellular Cmax values were 2.77 mg/L in the kidney and 1.21 mg/L in the spleen, whereas total interstitial Cmax values were 2.94 mg/L in the kidney and 3.01 mg/L in the spleen. Following Cmax, concentrations declined rapidly across plasma, interstitial fluid, and intracellular compartments; for instance, plasma levels decreased from 7.29 mg/L to 1.24 mg/L within 1 h after the second dose. Intracellular and interstitial trough concentrations Cmin remained below 0.1 mg/L at 120 h, indicating rapid clearance and the inability to maintain therapeutic levels under the simulated 5 mg/kg IV regimen. Dose-escalation simulations (5–100 mg/kg IV) demonstrated linear increases in exposure across the whole tested range.

**Figure 4:**
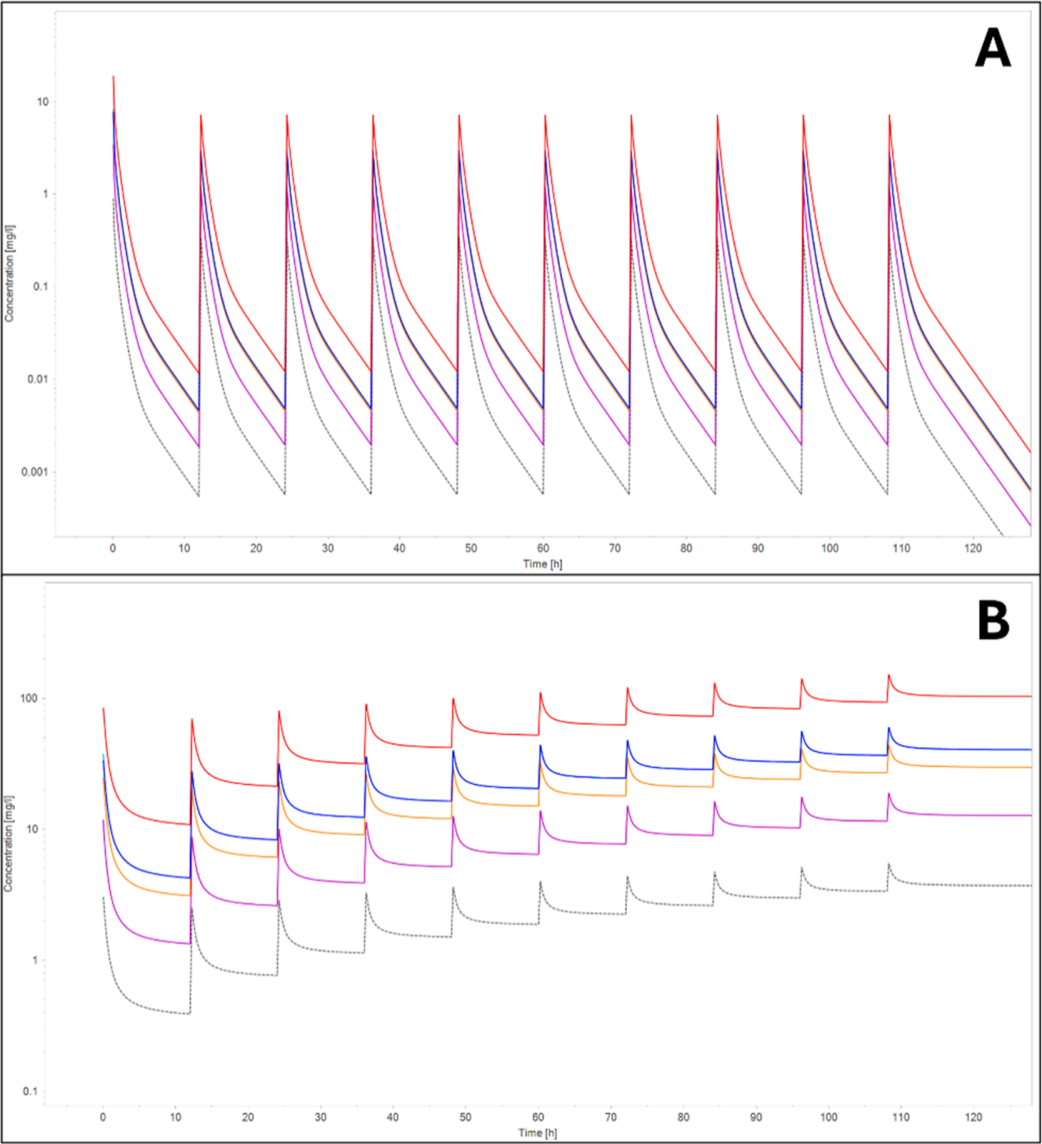
Simulated LMM6 concentrations in the plasma, interstitial, and intracellular compartments of kidney and spleen under the multiple-dose regimen (5 mg/kg IV q12h) in mice (A) and humans (700 mg or 10 mg/kg IV q12h) (B). *Orange: kidney intracellular total; purple: spleen intracellular total; blue: kidney interstitial total; gray dash: plasma venous unbound; red: plasma venous total. Additional Note: Spleen interstitial total concentration follows the same profile as kidney interstitial total.

In contrast, pharmacokinetic extrapolations to humans predicted a markedly different profile. For the 350 mg (5 mg/kg) q12h regimen (graph presented in the Supporting Information, Figure S2), the simulated plasma total Cmax after the second dose was approximately 35.00 mg/L (unbound fraction ≈1.26 mg/L). Predicted total intracellular Cmax values were 10.16 mg/L in the kidney and 13.97 mg/L in the spleen, while total interstitial Cmax values were 13.83 mg/L and 13.97 mg/L, respectively. From the sixth dose onward, LMM6 concentrations remained within the MIC range (8–32 mg/L) in the renal (intracellular and interstitial) and splenic (interstitial) compartments. Only the intracellular spleen compartment failed to reach the MIC threshold by the end of the 120 h simulation.

To achieve MIC-range concentrations earlier, a 700 mg (10 mg/kg) q12h regimen was simulated (Figure 4B). Under this higher-dose scenario, the predicted plasma total Cmax after the second dose was approximately 70.00 mg/L (unbound ≈2.52 mg/L). Predicted total intracellular Cmax values were 20.33 mg/L in the kidney and 8.80 mg/L in the spleen, whereas total interstitial Cmax values were 27.66 mg/L and 27.95 mg/L, respectively. With this regimen, LMM6 concentrations reached and maintained levels within the MIC range (8–32 mg/L) from the third dose onward in the renal (intracellular and interstitial) and splenic (interstitial) compartments, while the intracellular spleen compartment achieved these concentrations only after the seventh dose. Numerical details are available in the Supporting Information

## 4 Discussion

All measurements were supported by a validated HPLC assay that demonstrated high specificity and sensitivity (LOD = 0.28 mg/L, LOQ = 0.85 mg/L), excellent linearity (R^2^ = 0.998), and acceptable precision and accuracy.

The experimental data—a high melting point (222.5 °C), a positive logP (2.11), and consistently low thermodynamic solubility—indicate a hydrophobic compound with limited aqueous dissolution. Furthermore, the variation between logP replicates was less than 0.3 logarithmic units, which is consistent with acceptable criteria established in literature descriptions.^30,31^

Although solubility was modestly higher in acidic media than in neutral/alkaline conditions, it remained poor across all compendial and biorelevant media tested (Table 6 and Figure 3). These results suggest compatibility with a Biopharmaceutics Classification System designation at the boundary of classes II and IV, implying that the oral bioavailability of LMM6 is likely to be dissolution-limited and therefore dependent on formulation strategies;^42^ however, confirmation in preclinical studies is required.^8,43^

Solubility measured in biorelevant media (FaSSGF, FaSSIF, and FeSSIF) showed a small but measurable solubilizing effect relative to simple buffers. We interpret this effect as apparent solubilization mediated by bile salts and phospholipids (micellar/wetting phenomena) rather than an intrinsic change in aqueous solubility.^44,45^ Statistical analysis by ANOVA indicated that bile salt concentration significantly affected solubility (*p* = 1.09 × 10^–4^), but pH exerted a substantially larger influence (*p* = 3.03 × 10^–10^), underscoring the dominant role of ionization and pH-dependent dissolution in this compound’s behavior.

Interestingly, while the solubility of LMM6 in FeSSIF (fed state) was higher than that in FaSSIF v2 (fasted state) due to the higher bile salt concentration, the absolute increase was modest. This suggests that while nutritional status exerts an effect, it may not be sufficient to fully overcome the dissolution-limited absorption of LMM6 in vivo, reinforcing the need for enabling formulations, a predictive correlation supported by literature using biorelevant media.^26,46^

Although our in vitro biopharmaceutical characterization evaluated the potential for oral administration, PBPK simulations for the oral route (mouse and human) demonstrated negligible predicted fraction absorbed (F_abs_), likely due to the compound’s low solubility and consequent poor absorption. Independently, the primary PBPK simulations were conducted using an IV dosing regimen to directly replicate the in vivo efficacy protocol reported by Faria et al. However, for the mouse model, the simulated IV regimen (5 mg/kg q12h for 5 days) indicated that systemic and tissue exposures were insufficient to reach the in vitro MIC range (8–32 mg/L). This discrepancy may be explained by the fact that MIC values are determined in static, cell-free systems, which do not replicate the complexity of the in vivo environment, including critical factors such as plasma protein binding, immune responses, and localized tissue conditions like pH and oxygenation.^47–50^

When the in vitro MIC is considered as the target, both plasma and tissue compartments showed subtherapeutic levels, with rapid postdose declines and negligible trough concentrations at 120 h. This finding underscores an apparent paradox: if the in vivo efficacy reported in mice is valid, some hypothesis could be raised: it must be mediated by mechanisms not directly correlated with maintaining drug concentrations above the MIC; the actual tissue and plasma concentration should be higher than the predicted; and the tissue/plasma ratio assumed by the model needs to be confirmed and re-evaluated. On the basis of these questions, in vivo experiments need to be performed to explain the actual relationship between PK and pharmacodynamic data.

Besides these questions, the understanding of the dose feasibility for LMM6 in humans was investigated by the translatability of these findings. The simulations for human revealed a significantly different pharmacokinetic profile. At 350 mg (5 mg/kg) q12h, plasma and tissue concentrations were significantly higher than those predicted in mice, with total plasma Cmax ≈35 mg/L. Tissue exposures approached or reached the MIC range in most compartments after multiple doses. These results reflect interspecies differences in metabolic clearance and protein binding, leading to slower elimination and greater accumulation in humans.^51,52^ Notably, the model predicted that steady-state concentrations in the renal and splenic interstitial compartments were sustained within the in vitro MIC range after the sixth dose, indicating that repeated administration would maintain therapeutic levels at key sites of infection.

When the dose was doubled (700 mg/individual or 10 mg/kg q12h), predicted concentrations surpassed the MIC range in all evaluated compartments (Figure 4B), with earlier attainment of potentially efficacious exposures (from the third dose onward in renal and splenic interstitial spaces). These findings suggest a dose-dependent enhancement in tissue penetration and accumulation, particularly in the kidneys, where both interstitial and intracellular concentrations reached and maintained MIC levels early in the regimen. This distinction is clinically critical, as the pathogenesis of invasive fungal infections such as candidiasis involves both compartments: Candida spp. can reside extracellularly in interstitial fluid and, critically, persist intracellularly within host phagocytes, evading host defenses and complicating treatment.^53,54^

However, as depicted in Figure 4B, the progressive accumulation of LMM6 following multiple doses also raises concerns regarding potential systemic or tissue-specific toxicities. While this accumulation is necessary to achieve theoretical therapeutic efficacy, it highlights the critical need for future comprehensive toxicological studies to establish appropriate safety margins, ensuring that clinical regimens do not reach toxic thresholds in the human tissues.

Overall, the PBPK simulations suggest that human exposures at clinically feasible doses could achieve therapeutic levels, unlike those in mice, highlighting the importance of interspecies scaling for translational predictions and a promising outlook for the continuation of preclinical studies.

Nevertheless, model-based conclusions should be interpreted with caution. Quantitative predictions of intracellular and interstitial accumulation are highly sensitive to tissue partition coefficients (Kp), intracellular-to-extracellular unbound fractions, plasma–protein binding, and systemic clearance, many of which were estimated in silico or derived by interspecies scaling in the present work. In addition, the identity and contribution of specific metabolic enzymes remain uncertain: the model’s metabolic pathways were based on in silico CYP predictions, which adds a further layer of uncertainty to the clearance estimates.^55–57^ As emphasized, these parameters constitute the principal sources of uncertainty; therefore, empiric measurement of plasma–protein binding, direct tissue or cellular concentration determinations, experimentally derived Kp values, and metabolite identification/quantification are essential to validate and refine the models. Despite these uncertainties, the present PBPK framework provides a robust basis for hypothesis generation and dose optimization in forthcoming experimental and clinical evaluations.

From a development perspective, two strategic implications follow. First, given the limited aqueous solubility and negligible *F*_abs_, as well as the reliance on the intravenous route in preclinical efficacy studies, prioritizing an IV formulation program for early stage PK/PD and toxicology studies is justified. IV administration bypasses the dissolution-limited absorption barrier, enabling a direct assessment of systemic and tissue exposure, as well as safety margins.^58–60^ Second, if oral delivery is pursued, formulation strategies that enhance apparent solubility—such as lipid-based systems, amorphous solid dispersions, or nanoparticle/micellar approaches—should be investigated, accompanied by in vitro–in vivo correlation experiments in fed and fasted states to evaluate practical benefit.^61–63^ The measured log P (∼2.1) suggests that permeability is unlikely to be limiting, as this value falls within the optimal range for passive membrane diffusion described in the literature;^6,42^ therefore, improving dissolution should be the principal focus of oral formulation efforts.

Finally, by integrating targeted experimental measurements into the PBPK workflow, we enhanced the physiological realism of our simulations and reduced the reliance on purely in silico estimates for LMM6. Although the lack of actual in vivo pharmacokinetic data represents a limitation of the present study, it reflects the early preclinical stage of this candidate. Rather than a definitive dosing guide, this established model provides a rational framework for hypothesis generation and experimental prioritization—such as optimizing future in vivo protocols and sampling time points. This step is essential to derisk the translational development of this candidate.

## 5 Conclusion

The integration of experimental physicochemical characterization of antifungal LMM6 with PBPK (MID3) modeling was essential for translating early findings into development strategies. Assays confirmed that LMM6 is a hydrophobic compound (log P 2.11) with low thermodynamic solubility across all tested media, suggesting that its oral absorption would be dissolution-limited. The solubility profile revealed that pH exerted a significantly greater influence than the bile salt concentration.

Critically, the PBPK model revealed a key insight: the observed in vivo efficacy in mice could not be totally explained by low systemic (plasma) or general tissue exposures, which remained subtherapeutic relative to the MIC. This apparent paradox highlights the inherent limitations of relying solely on purely pharmacokinetic parameters or the MIC, determined in cell-free systems, to predict efficacy in an immunocompetent host where immune components and the tissue microenvironment play a crucial role.

The PBPK extrapolation to humans, however, predicts a considerably more favorable pharmacokinetic profile. The predicted ability of the compound to reach therapeutic concentrations in both interstitial and intracellular compartments suggests a higher potential for infection eradication in humans compared to that observed in mice.

Strategically, this necessitates prioritizing an IV formulation for early PK and/or PD studies to bypass the dissolution barrier. If the oral route is pursued, formulation efforts must concentrate on strategies that enhance dissolution, as permeability is less likely to be limiting. In addition to these insights, this study provides a framework for protocol design in in vivo investigations. Since quantitative tissue predictions depend on in silico ADME estimates, the model should be used as a tool to prioritize future experiments, including empirical measurements of plasma-protein binding, metabolic clearance, and tissue partition coefficients (Kp), to refine LMM6’s translational profile.

## Supplementary Material



## Notes

Detailed solubility, ANOVA, and PBPK simulation data; theoretical solubility predictions (in silico) in water and biorelevant media; chromatogram profile of the LMM6 standard solution in HPLC with a UV–vis detector at 273 nm; detailed linear regression and ANOVA analysis results for the effects of pH and bile salt concentration on solubility; simulated LMM6 concentrations in plasma and tissue under multiple-dose regimen (5 mg/kg IV q12h) in human; numerical predicted pharmacokinetic profiles of LMM6 in mouse plasma and tissues across multiple-dose regimens (5 mg/kg q12h); and numerical predicted pharmacokinetic profiles of LMM6 in human plasma and tissues across multiple-dose regimens (5 and 10 mg/kg q12h) (PDF)

## Abbreviations

PBPK: physiologically based pharmacokinetic
MID3: Model-Informed Drug Discovery and Development
LMM6: code name for the 1,3,4-oxadiazole derivative
FaSSGF: Fasted State Simulated Gastric Fluid
FaSSIF: Fasted State Simulated Intestinal Fluid
FeSSIF: Fed State Simulated Intestinal Fluid
logP: logarithm of the partition coefficient (lipophilicity)
HPLC: high-performance liquid chromatography
LOD: limit of detection
LOQ: limit of quantification
ANOVA: analysis of variance
IV: intravenous
q12: every 12 h (dosing frequency)
*F*abs: fraction absorbed
*C*max: maximum (peak) concentration
*C*min: minimum concentration
MIC: minimum inhibitory concentration
PK/PD: pharmacokinetics/pharmacodynamics
Kp: tissue partition coefficient
rpm: rotation per minute
