# Supplementary material for: Integrating Experimental Physicochemical Data with PBPK Modeling to Guide the Translational Development of the Antifungal 1,3,4-Oxadiazole Derivative LMM6
Source: ACS Omega. 2026 Jul 2;11(28):41468–78. doi: 10.1021/acsomega.6c00258 (PMC13393369; doi:10.1021/acsomega.6c00258)
Supplement: Supplementary file 1 [file ao6c00258_si_001.pdf]

INTEGRATING EXPERIMENTAL PHYSICOCHEMICAL DATA WITH PBPK MODELING TO GUIDE THE TRANSLATIONAL DEVELOPMENT OF THE ANTIFUNGAL 1,3,4-OXADIAZOLE DERIVATIVE LMM6

João M. B. Piai[a], *Victor N. M. Lippa*[a]; *Edilainy R. Caleffi-Marchesini*[b]; *Fernanda B. B. Pangoni*[a], *Maria S. Felipe*[c]; *Erika S. Kioshima*[a]; *Andrea Diniz*[a*].

[a] State University of Maringá (UEM), Maringá, Paraná- Brazil

[b] Ingá University Center (UNINGÁ), Maringá, Paraná- Brazil

[c] Catholic University of Brasilia and University of Brasília, Brasília, Federal District - Brazil

SUPPLEMENTARY MATERIAL

Table S1. Theoretical Solubility of LMM6 (Using ADMET Predictor® v.11).

Media	Estimated solubility (mg/L)
**Water**	2
**FaSSGF**	47
**FaSSIF**	10
**FeSSIF**	82

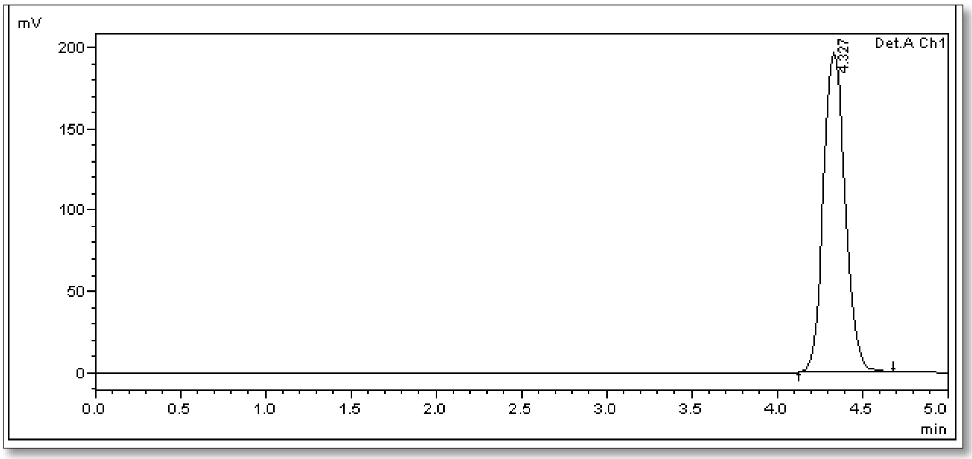

Figure S1. Chromatogram profile of the LMM6 standard solution in HPLC with a UV-VIS detector at 273 nm.

Table S2. Linear regression analysis results for correlation between solubility in biorelevant media and pH values and solubility and bile salt concentrations.

**pH effect**						
Regression analysis						
R	0.934					
Rsq	0.873					
Rsq_{adj}	0.865					
SE	0.803					
n	18					
ANOVA						
	GL	*SQ*	*MQ*	*F*	*Significance F*	
Regression	1	71.351	71,351	110.47	1.37E-08	
Resíduo	16	10.333	0.6458			
Total	17	81.685				
	Coefficients	*SE*	*Stat t*	*P-value*	*95% -lower*	*95% superior*
Interception	13.275	0.832	15.949	3.03E-11	11.510	15.039
pH	-2.301	0.219	-10.510	-2.76621	-2.766	-1.837
**Bile salt concentration effect**						
Regression analysis						
R	0.842					
Rsq	0.709					
Rsq_{adj}	0.668					
SE	5.86E-06					
n	9					
ANOVA						
	GL	*SQ*	*MQ*	*F*	*Significance F*	
Regression	1	5.86E-10	5.86E-10	17.06986	0.00439	
Resíduo	7	2.4E-10	3.43E-11			
Total	8	8.27E-10				
	Coefficients	*SE*	*Stat t*	*P-value*	*95% -lower*	*95% superior*
Regression	2.32E-05	2.98E-06	7.783	0.000109	1.61E-05	3.03E-05
Resíduo	-2.64E-06	6.4E-07	-4.131	0.004395	-4.15E-06	-1.1E-06
Total	2.32E-05	2.98E-06	7.783	0.000109	1.61E-05	3.03E-05

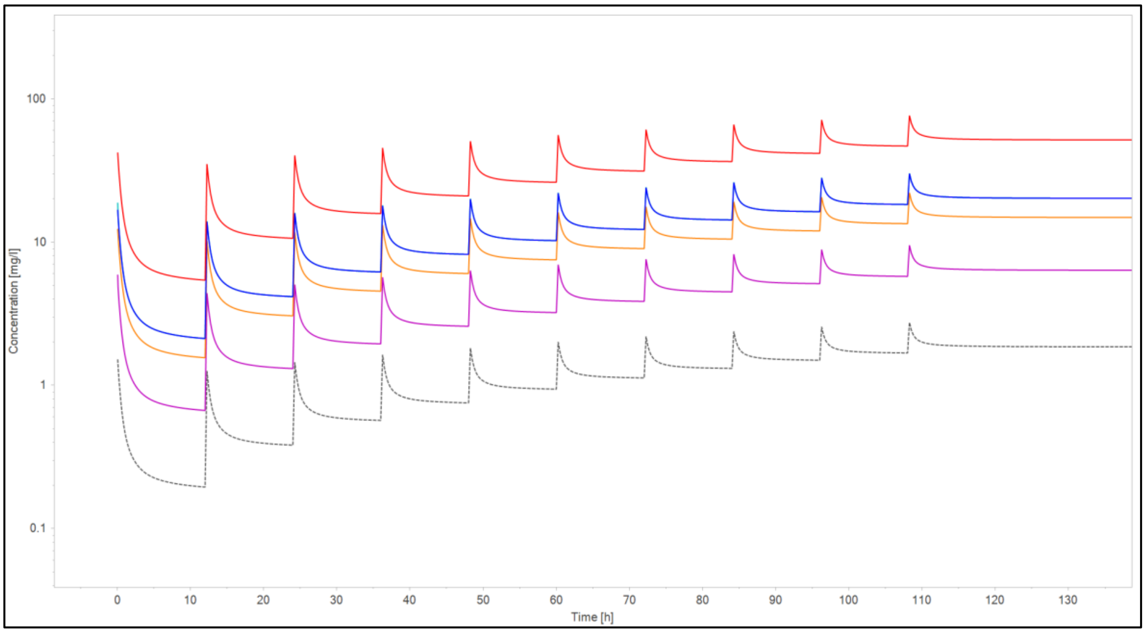

Figure S2. Simulated LMM6 concentrations in plasma, interstitial, and intracellular compartments of kidney and spleen under multiple-dose regimen 350 mg (5 mg/kg IV q12h) in human.

**Orange: kidney intracellular total; purple: spleen intracellular total; blue: kidney interstitial total; gray dash: plasma venous unbound; red: plasma venous total. Additional Note: Spleen interstitial total concentration follows the same profile as Kidney interstitial total.*

Table S3. Predicted Pharmacokinetic Profiles of LMM6 in Mouse Plasma and Tissues from a PK-Sim Simulation of a 5 mg/kg q12h Multiple-Dose Regimen.

TIME [h]	Venous Blood-Plasma Total Concentration [mg/L]	Venous Blood-Plasma Unbound Conc. [mg/L]	Kidney Interstitial Total Conc. [mg/L]	Kidney Interstitial Unbound Conc. [mg/L]	Kidney Intracellular Total Conc. [mg/L]	Kidney Intracellular Unbound Conc. [mg/L]	Spleen Interstitial Total Conc. [mg/L]	Spleen Interstitial Unbound Conc.[mg/L]	Spleen Intracellular Total Conc. [mg/L]	Spleen Intracellular Unbound Conc. [mg/L]
0.05	19.177	0.901	7.879	7.879	7.438	7.438	8.466	8.466	3.423	3.423
1	1.735	0.082	0.694	0.694	0.655	0.655	0.701	0.701	0.283	0.283
6	0.055	0.003	0.022	0.022	0.020	0.020	0.022	0.022	0.009	0.009
12	0.012	0.001	0.005	0.005	0.004	0.004	0.005	0.005	0.002	0.002
12.25	7.300	0.343	2.941	2.941	2.776	2.776	3.011	3.011	1.217	1.217
24	0.012	0.001	0.005	0.005	0.005	0.005	0.005	0.005	0.002	0.002
24.25	7.300	0.343	2.941	2.941	2.776	2.776	3.011	3.011	1.217	1.217
36	0.012	0.001	0.005	0.005	0.005	0.005	0.005	0.005	0.002	0.002
36.25	7.300	0.343	2.941	2.941	2.776	2.776	3.011	3.011	1.217	1.217
48	0.012	0.001	0.005	0.005	0.005	0.005	0.005	0.005	0.002	0.002
48.25	7.300	0.343	2.941	2.941	2.776	2.776	3.011	3.011	1.217	1.217
60	0.012	0.001	0.005	0.005	0.005	0.005	0.005	0.005	0.002	0.002
60.25	7.300	0.343	2.941	2.941	2.776	2.776	3.011	3.011	1.217	1.217
72	0.012	0.001	0.005	0.005	0.005	0.005	0.005	0.005	0.002	0.002
72.25	7.300	0.343	2.941	2.941	2.776	2.776	3.011	3.011	1.217	1.217
84	0.012	0.001	0.005	0.005	0.005	0.005	0.005	0.005	0.002	0.002
84.25	7.300	0.343	2.941	2.941	2.776	2.776	3.011	3.011	1.217	1.217
96	0.012	0.001	0.005	0.005	0.005	0.005	0.005	0.005	0.002	0.002
96.25	7.300	0.343	2.941	2.941	2.776	2.776	3.011	3.011	1.217	1.217
108	0.012	0.001	0.005	0.005	0.005	0.005	0.005	0.005	0.002	0.002
108.25	7.300	0.343	2.941	2.941	2.776	2.776	3.011	3.011	1.217	1.217
120	0.012	0.001	0.005	0.005	0.005	0.005	0.005	0.005	0.002	0.002

Table S4. Predicted Pharmacokinetic Profiles of LMM6 in Human Plasma and Tissues from a PK-Sim Simulation of a 5 mg/kg q12h Multiple-Dose Regimen.

TIME [h]	Venous Blood-Plasma Total Concentration [mg/L]	Venous Blood-Plasma Unbound Conc. [mg/L]	Kidney Interstitial Total Conc. [mg/L]	Kidney Interstitial Unbound Conc. [mg/L]	Kidney Intracellular Total Conc. [mg/L]	Kidney Intracellular Unbound Conc. [mg/L]	Spleen Interstitial Total Conc. [mg/L]	Spleen Interstitial Unbound Conc.[mg/L]	Spleen Intracellular Total Conc. [mg/L]	Spleen Intracellular Unbound Conc. [mg/L]
**0.05**	42.155	1.518	16.795	16.795	12.354	12.354	18.794	18.794	5.919	5.919
**1**	13.395	0.482	5.268	5.268	3.872	3.872	5.296	5.296	1.667	1.667
**6**	6.033	0.217	2.361	2.361	1.735	1.735	2.362	2.362	0.744	0.744
**12**	5.418	0.195	2.120	2.120	1.558	1.558	2.121	2.121	0.668	0.668
**12.25**	35.002	1.260	13.831	13.831	10.167	10.167	13.979	13.979	4.401	4.401
**24**	10.620	0.382	4.156	4.156	3.054	3.054	4.156	4.156	1.309	1.309
**24.25**	40.203	1.447	15.866	15.866	11.663	11.663	16.014	16.014	5.042	5.042
**36**	15.796	0.569	6.182	6.182	4.542	4.542	6.182	6.182	1.946	1.946
**36.25**	45.379	1.634	17.891	17.891	13.151	13.151	18.040	18.040	5.680	5.680
**48**	20.963	0.755	8.203	8.203	6.028	6.028	8.204	8.204	2.583	2.583
**48.25**	50.546	1.820	19.913	19.913	14.636	14.636	20.061	20.061	6.316	6.316
**60**	26.124	0.940	10.223	10.223	7.512	7.512	10.224	10.224	3.219	3.219
**60.25**	55.707	2.005	21.933	21.933	16.120	16.120	22.081	22.081	6.952	6.952
**72**	31.281	1.126	12.241	12.241	8.994	8.994	12.242	12.242	3.854	3.854
**72.25**	60.864	2.191	23.951	23.951	17.603	17.603	24.099	24.099	7.588	7.588
**84**	36.435	1.312	14.258	14.258	10.476	10.476	14.258	14.258	4.489	4.489
**84.25**	66.017	2.377	25.968	25.968	19.085	19.085	26.116	26.116	8.222	8.222
**96**	41.585	1.497	16.273	16.273	11.957	11.957	16.274	16.274	5.124	5.124
**96.25**	71.168	2.562	27.983	27.983	20.566	20.566	28.131	28.131	8.857	8.857
**108**	46.734	1.682	18.288	18.288	13.437	13.437	18.288	18.288	5.758	5.758
**108.25**	76.316	2.747	29.998	29.998	22.046	22.046	30.146	30.146	9.491	9.491
**120**	51.881	1.868	20.302	20.302	14.917	14.917	20.303	20.303	6.392	6.392

Table S5. Predicted Pharmacokinetic Profiles of LMM6 in Human Plasma and Tissues from a PK-Sim Simulation of a 10 mg/kg q12h Multiple-Dose Regimen.

TIME [h]	Venous Blood-Plasma Total Concentration [mg/L]	Venous Blood-Plasma Unbound Conc. [mg/L]	Kidney Interstitial Total Conc. [mg/L]	Kidney Interstitial Unbound Conc. [mg/L]	Kidney Intracellular Total Conc. [mg/L]	Kidney Intracellular Unbound Conc. [mg/L]	Spleen Interstitial Total Conc. [mg/L]	Spleen Interstitial Unbound Conc.[mg/L]	Spleen Intracellular Total Conc. [mg/L]	Spleen Intracellular Unbound Conc. [mg/L]
0.05	84.310	3.036	33.590	33.590	24.708	24.708	37.588	37.588	11.838	11.838
1	26.790	0.964	10.536	10.536	7.744	7.744	10.592	10.592	3.335	3.335
6	12.066	0.434	4.722	4.722	3.470	3.470	4.724	4.724	1.487	1.487
12	10.836	0.391	4.241	4.241	3.116	3.116	4.242	4.242	1.335	1.335
12.25	70.005	2.520	27.662	27.662	20.334	20.334	27.958	27.958	8.802	8.802
24	21.241	0.764	8.313	8.313	6.107	6.107	8.312	8.312	2.617	2.617
24.25	80.407	2.895	31.733	31.733	23.326	23.326	32.028	32.028	10.084	10.084
36	31.592	1.139	12.364	12.364	9.084	9.084	12.364	12.364	3.892	3.892
36.25	90.758	3.268	35.783	35.783	26.303	26.303	36.080	36.080	11.359	11.359
48	41.926	1.510	16.406	16.406	12.056	12.056	16.409	16.409	5.166	5.166
48.25	101.092	3.640	39.827	39.827	29.273	29.273	39.930	39.930	12.632	12.632
60	52.249	1.881	20.446	20.446	15.024	15.024	20.448	20.448	6.437	6.437
60.25	111.414	4.179	43.866	43.866	32.240	32.240	44.162	44.162	13.904	13.904
72	62.562	2.252	24.482	24.482	17.987	17.987	24.484	24.484	7.708	7.708
72.25	121.728	4.508	47.902	47.902	35.206	35.206	48.198	48.198	15.175	15.175
84	72.869	2.623	28.515	28.515	20.952	20.952	28.515	28.515	8.978	8.978
84.25	132.034	4.754	51.936	51.936	38.170	38.170	52.232	52.232	16.445	16.445
96	83.170	3.001	32.546	32.546	23.913	23.913	32.548	32.548	10.247	10.247
96.25	142.336	5.125	55.966	55.966	41.132	41.132	56.238	56.238	17.714	17.714
108	93.468	3.364	36.576	36.576	26.874	26.874	36.576	36.576	11.516	11.516
108.25	152.632	5.494	59.996	59.996	44.092	44.092	60.292	60.292	18.983	18.983
120	103.760	3.736	40.604	40.604	29.834	29.834	40.606	40.606	12.784	12.784
